# Light people: professor Fei Ding

**DOI:** 10.1038/s41377-025-02043-1

**Published:** 2025-09-28

**Authors:** Siqiu Guo

**Affiliations:** 1 Light Publishing Group, Changchun Institute of Optics, Fine Mechanics and Physics, Chinese Academy of Sciences, Changchun, China; 2 Ji Hua Laboratory, Foshan, China

## Abstract

A Journey from Laboratories in China to Germany’s prestigious Leibniz University Hannover, where a visionary scientist is shaping the future of semiconductor materials and quantum photonic devices.

With an accomplished academic background spanning China, Germany, the Netherlands, and Switzerland, and currently serving as Chair Professor at Leibniz University Hannover, Professor Fei Ding leads his team in developing scalable and practical quantum technologies. His distinguished career is further highlighted by his reception of the prestigious ERC Starting Grant, Consolidator Grant, and Proof-of-Concept Grant.

In this issue of Light People, we are honored to feature this exceptional talent—Professor Fei Ding—and explore together the journey of his inspiring and highly accomplished career.

## Graphical abstract

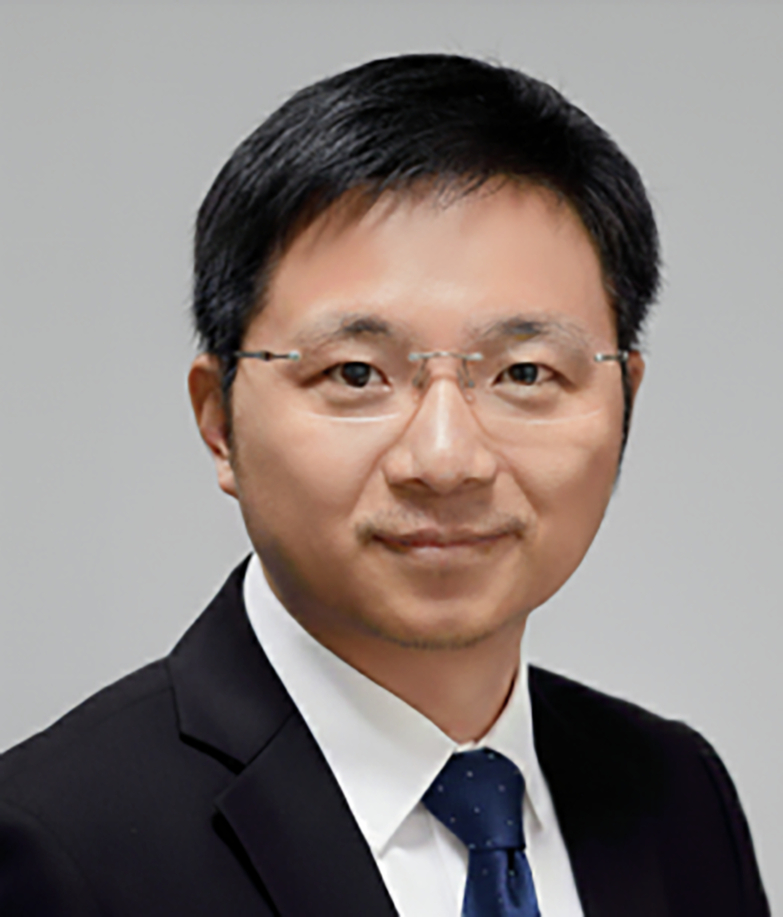


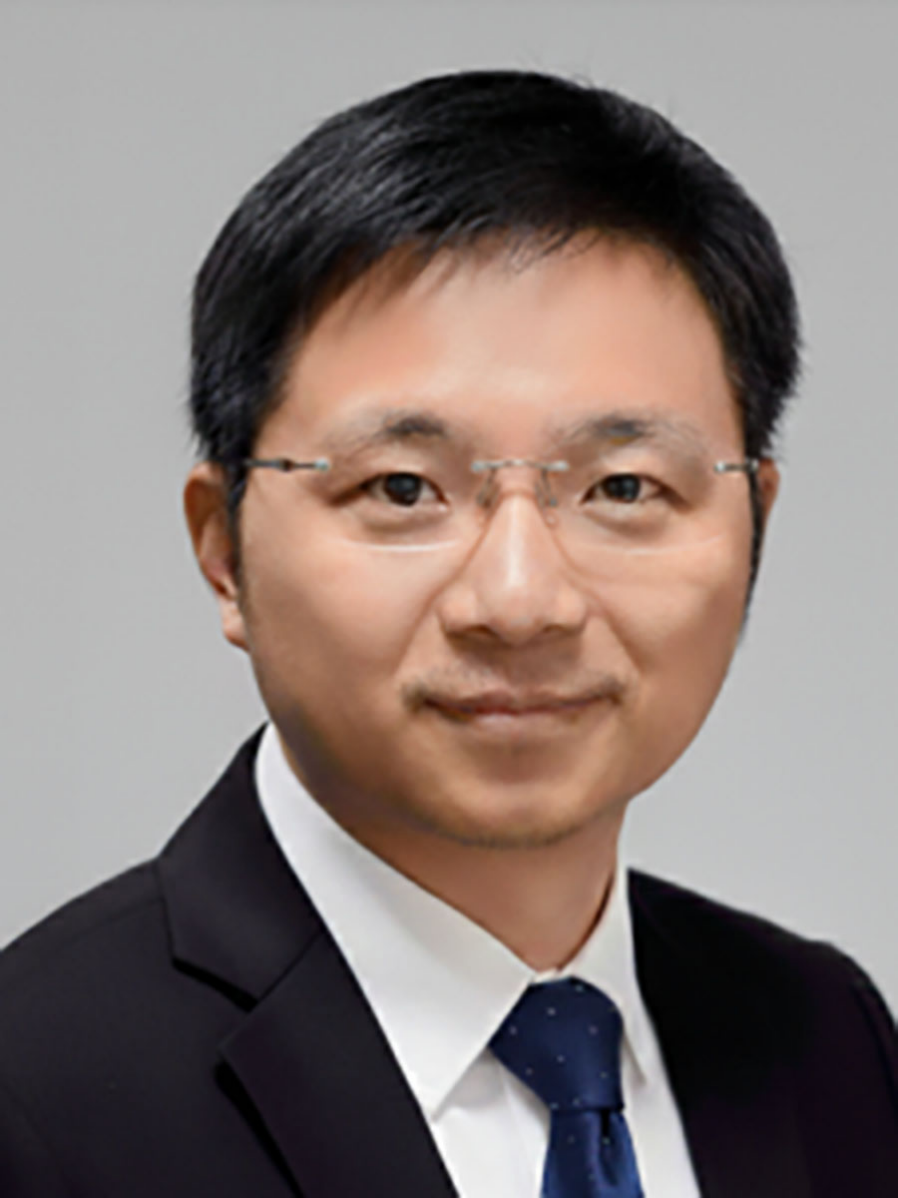
**Short Bio:** Fei Ding is a full professor (W3 Chair in Nanophysics) at Leibniz University Hannover in Germany. Prof. Ding received BSc degree in 2003 from Hefei University of Technology. In 2009 he obtained PhD degree from the joint doctoral promotion program between Max Planck Society Germany and Chinese Academy of Sciences. From 2010 to 2012 he was a Marie Curie Fellow at IBM Zurich Research Laboratory. In 2012, he became a group leader in IFW Dresden and then in 2016 moved to Hannover as a full professor. He is a recipient of the prestigious ERC Starting grant, Consolidator grant and Proof-of-Concept grant.

**1. As an expert in semiconductor materials and optical devices, could you systematically introduce your current core research directions?**

Our research is centered around semiconductor materials and quantum photonic devices, with the overarching goal of enabling scalable and practical quantum technologies. Broadly speaking, there are three interconnected directions, see also Fig. 1:

- *Solid-state quantum emitters*: We develop low-noise, deterministic single-photon and entangled-photon sources based on semiconductor materials. This includes epitaxial growth techniques and photonic integration schemes to push the limits of quantum light emissions.
- *Photonics with semiconductor nanostructures*: We design and fabricate novel nanophotonic structures, such as waveguides, cavities, and photonic crystals, to manipulate light–matter interactions at the nanoscale. These devices provide the backbone for efficient light generation, guiding, and detection.
- *Quantum networks and metrology*: Building on these materials and photonic devices, we explore applications in quantum communication and quantum metrology networks. For example, our recent project from European Research Council (ERC) “MiNet” explores large-scale multipartite entanglement distribution and synchronization of quantum communication links across cities, paving the way for future quantum internet applications.

**Figure:**
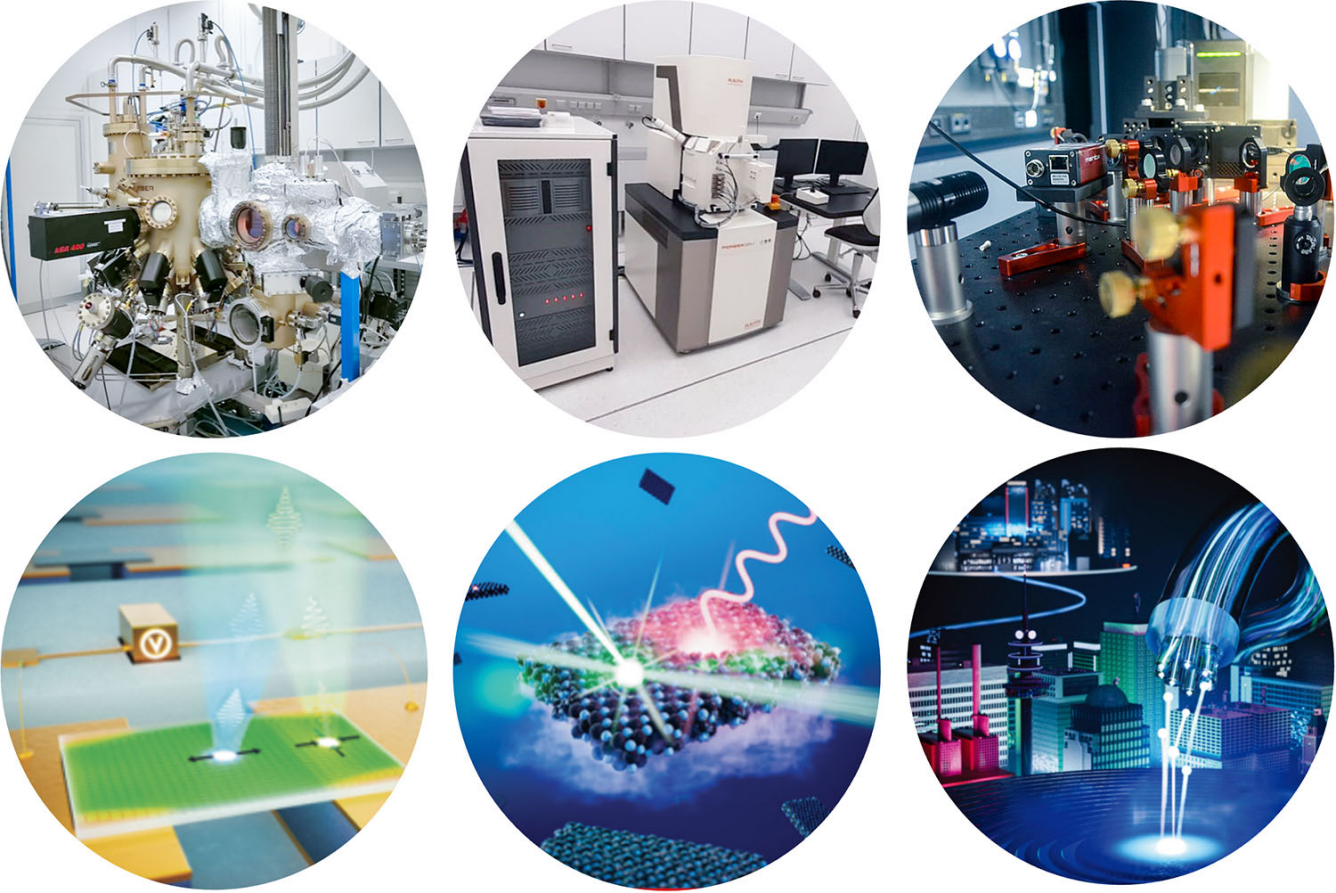
**Figure 1 (Upper row) Some of the research facilities in Prof. Fei Ding’s group, including material growth, optical device fabrication, and the quantum optics laboratory. (Bottom row) A selection of recent research results published as cover highlights**

**2. In 2024, you published a paper “High-rate intercity quantum key distribution with a semiconductor single-photon source**^1^**” in** ***Light: Science and Applications (Light)*. Could you briefly walk us through the key breakthroughs achieved in this work?**

In this work, we showed for the first time that a semiconductor single-photon source can enable high-rate quantum key distribution between two cities. Together with colleagues from University of Stuttgart (Prof. Peter Michler) and PTB (Prof. Stefan Kück), we generated single photons with excellent purity, high repetition rates and brightness, which are essential for secure quantum communication. Instead of a simple lab test, we deployed the system on the real fiber network connecting Hannover and Braunschweig, about 79 kilometers apart, and managed to achieve one of the highest secret key rates ever reported by using single photons. This result demonstrates that semiconductor quantum light sources are not just a laboratory concept, but a realistic technology for future quantum internet infrastructure. This is also the backbone for our recently funded ERC Proof-of-Concept project “ComPQT”, see Fig. 2.

**Figure:**
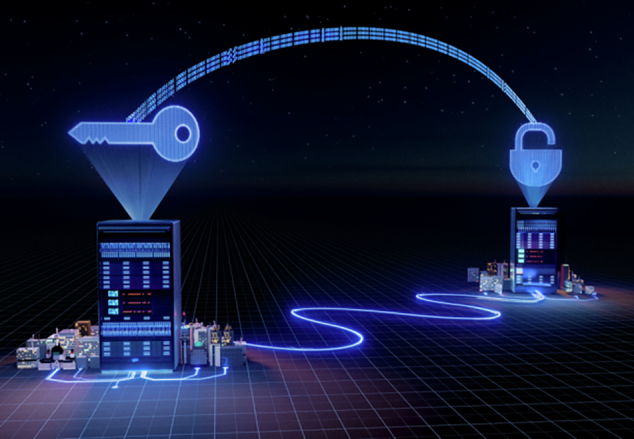
**Figure 2 The ERC Proof-of-Concept project ComPQT will develop compact, plug-and-play network terminals based on genuinely deterministic quantum light sources. These terminals will be tested over a 79-kilometre quantum link between Hannover and Braunschweig, demonstrating real-time quantum key distribution with ultra-precise time and frequency synchronization**

**3. Given your significant achievements in semiconductor-based quantum networks, what emerging trends do you foresee as most critical for the field’s development?**

Looking ahead, I see a few critical challenges that need to be addressed to really move semiconductor-based quantum networks forward^2^: First, turning high quality solid-state quantum emitters into fully telecom-ready, cavity-enhanced, indistinguishable single- and entangled-photon sources with deterministic coupling and active electrical tuning. This will push the quality of quantum light sources to the “system-grade” level rather than just lab records; Second, hybrid integration with photonic chips will be important. The excellent sources need to be combined with on-chip components, such as filters, switches and frequency converters, and tied to cryo-CMOS control and fast feed-forward/multiplexing, so we can increase rates of photon processing without sacrificing fidelity; third, but maybe the most challenging one, interfaces to quantum memories and spin-photon nodes to enable repeater-style architectures for optical quantum computing and also entanglement swapping over long distances; and finally, network engineering at large scale. This includes, but not limited to, phase/time-frequency stabilization on deployed fiber, protocol choices like MDI/TF-QKD with solid-state sources, and ultrafast optoelectronics.

**4. As an accomplished scholar with academic backgrounds in China, Germany, Netherland, and Switzerland, currently serving as Chair Professor at Leibniz University Hannover, how has this transcultural academic leadership experience fundamentally shaped your approach to building research teams and formulating technology roadmaps?**

That’s a very important question. Having had the chance to study and work in groups in different countries, I’ve learned that each academic culture and research topic brings their own strengths. This is also why I strongly encourage young researchers to look around and to broaden their horizons early in their careers. These experiences, for example in material growth, photonic device and quantum optics, have fundamentally shaped the way I build my own team. When it comes to technology roadmaps, as I learnt from my previous supervisors, I believe in setting bold, long-term goals, like scalable quantum networks, but breaking them down into concrete, achievable steps. After all, *per aspera ad astra*, only through hard challenges can we reach the stars.

**5. Could you share with us what the biggest challenge you encountered was on your journey from studying in Germany to becoming a professor, and how did you overcome these challenges?**

One of the biggest challenges for me, after coming to Europe, was adapting to a completely different academic culture. The expectations on independence and critical thinking were quite different from what I was used to. For example, back in China I tended to wait for very concrete instructions from my supervisor, and I have weekly report to him in detail. In the lab, I dared to try new things on my own, because I worry that I might damage expensive equipment. In Germany, however, my supervisor only outlined his ideas in broad terms, gave me a thorough introduction to the equipment, and then left it entirely to me. When I first faced a new laser, he simply reminded me of the safety rules and encouraged me to try it. Through this trial and error, I not only learned to solve problems independently but also discovered efficient approaches.

Looking back to this journey, I think what helped me overcome the “culture shock” was persistence and openness to learn from my supervisors. Today, I think I have learnt to turn cultural differences into strengths. I feel those challenges when I was facing as a student were gifts, which shaped me into an established Chinese professor in Germany.

**6. Having been awarded the prestigious ERC grant three times, in 2016, 2022 and 2025, what would you identify as the most critical elements young researchers should focus on when preparing competitive funding applications?**

Successful funding application is crucial to the career of young researchers. From my experience, the most critical elements for preparing a competitive funding application are clarity of ambitious vision, a well-structured research plan, balance between risk and gain, and demonstrating possible future impact. Many young researchers are not properly trained in funding writing. They can learn from their supervisors/peers on how to clearly describe the research question, why it matters, and how their approach is innovative. It is worth mentioning that many funding schemes, such as the EU programs, want to see the potential broader impact, which means not just high-impact papers, but also impact for technology, society, or industry.

**7. As an editorial board member of** ***Light*, what strategic approaches do you believe the journal should adopt to maintain its distinctive characteristics and enhance its competitive advantages in building a high-quality journal?**

I think *Light* is already doing an excellent job, so here I can only echo some of the strengths I see in the journal. To maintain its distinctive character (bridging science and applications) and strengthen its competitiveness, it should continue focusing on high-quality, cutting-edge research while encouraging interdisciplinary works in broader areas related to optics and photonics. Another key is rapid yet rigorous peer review. This will ensure timely publication without compromising standards and gain the trust from authors. Then, *Light* can enhance its impact by establishing international offices, actively highlighting emerging trends (where I also have been contributing my efforts), promoting special issues on hot topics. Finally, fostering a global community of authors and readers should be also an important task of Light. As the general co-Chair of the Light Conference 2025, I witnessed a successful event of how bringing together researchers from different regions. It strengthens the journal’s role as a hub for high-quality, interdisciplinary research.
